# Circadian rhythms and renal pathophysiology

**DOI:** 10.1172/JCI148277

**Published:** 2022-02-01

**Authors:** Rajesh Mohandas, Lauren G. Douma, Yogesh Scindia, Michelle L. Gumz

**Affiliations:** 1 Department of Medicine, Division of Nephrology,; 2 Center for Integrative Cardiovascular and Metabolic Diseases,; 3 Department of Biochemistry and Molecular Biology,; 4 Department of Medicine, Division of Pulmonary, Critical Care, and Sleep Medicine,; 5 Department of Pathology, and; 6 Department of Physiology and Functional Genomics, University of Florida, Gainesville, Florida, USA.

## Abstract

The reality of life in modern times is that our internal circadian rhythms are often out of alignment with the light/dark cycle of the external environment. This is known as circadian disruption, and a wealth of epidemiological evidence shows that it is associated with an increased risk for cardiovascular disease. Cardiovascular disease remains the top cause of death in the United States, and kidney disease in particular is a tremendous public health burden that contributes to cardiovascular deaths. There is an urgent need for new treatments for kidney disease; circadian rhythm–based therapies may be of potential benefit. The goal of this Review is to summarize the existing data that demonstrate a connection between circadian rhythm disruption and renal impairment in humans. Specifically, we will focus on chronic kidney disease, lupus nephritis, hypertension, and aging. Importantly, the relationship between circadian dysfunction and pathophysiology is thought to be bidirectional. Here we discuss the gaps in our knowledge of the mechanisms underlying circadian dysfunction in diseases of the kidney. Finally, we provide a brief overview of potential circadian rhythm–based interventions that could provide benefit in renal disease.

## Introduction

Renal function is essential to maintaining systemic homeostasis. The kidneys filter the blood, maintain body pH, water, and electrolyte balance, and contribute to the regulation of blood pressure. The kidneys produce urine as a byproduct of maintaining fluid and electrolyte balance. The volume and composition of the urine can provide insight into health status. Indeed, analysis of the urine is thought to be the oldest medical test, dating back to the ancient Babylonians and Sumerians who recorded their observations on clay tablets more than 6000 years ago (1). This practice was termed “uroscopy,” meaning the scientific examination of urine. Thousands of years later, Hippocrates practiced uroscopy and believed that changes in the properties of urine were indicative of disease states. For example, from this time through the Middle Ages, medical practitioners would taste urine in order to detect sweetness, which was indicative of diabetes mellitus. In the 11th century, the physician Ismail al-Jurjani noted that urine composition could be altered by food intake as well as by aging. Interestingly, Ismail purportedly asked for a full 24-hour collection of urine and emphasized the importance of a good night’s sleep prior to the collection. This report provides perhaps the earliest hint at the incorporation of time as a biological variable in the practice of medicine.

It was documented as early as the end of the 19th century (reviewed in ref. 2) that urine volume varies over the course of a 24-hour period, although this observation was largely attributed to differences in fluid intake in the active versus the rest phase. In the 1950s, landmark studies by Mills and colleagues firmly established the rhythmicity of renal function (3–5). These investigators showed that urinary sodium, potassium, chloride, and phosphate were excreted with a 24-hour rhythmic pattern and that urine volume and pH varied over the 24-hour cycle as well. Importantly, these experiments were conducted under relatively constant conditions so that fluid and food intake, posture, and sleep stage were all controlled. Thus, it could be concluded that the rhythmic variations were endogenous and not a result of behavior or posture. In the decades since, enormous strides have been made in understanding the molecular mechanisms underlying rhythmic renal function. The discovery of circadian clock genes, for which Hall, Rosbash, and Young were awarded the Nobel Prize in 2017, made it possible to study the molecular mechanisms of circadian rhythms (6).

**Figure 1:**
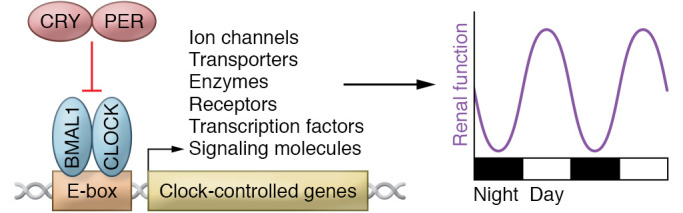
Transcription-translation feedback loop of the circadian clock. BMAL1 and CLOCK bind to E-box response elements in the promoters of target genes, which include Period and Cryptochrome. PER and CRY form the negative arm of this feedback loop. Ancillary loops of the transcription-translation feedback system involving nuclear receptors and posttranslational modifications exist but will not be discussed here. Also beyond the scope of this Review is a discussion of the non-canonical functions of clock proteins, such as the role of BMAL1 in the regulation of translation in the cytosol (161).

The circadian system is conducted by the central clock, located in the suprachiasmatic nucleus of the hypothalamus, through neuronal and humoral signaling to the “orchestra” of the body’s peripheral clocks, which include other regions of the brain and tissue clocks such as the kidney. Indeed, the molecular machinery of the circadian clock is present in nearly every cell and tissue type in humans, and its mechanisms have been extensively reviewed (7, 8). Briefly, the core components of the clock comprise transcription factors that function in a transcription-translation feedback loop (Figure 1). In the positive arm, BMAL1 and CLOCK proteins heterodimerize and bind E-box response elements in the promoters of target genes. These target genes include those encoding the PER and CRY proteins that function in the negative arm of the feedback system. PER and CRY feed back on and inhibit the activity of BMAL1 and CLOCK, thus decreasing the transcription of their own genes in addition to other target genes. This mechanism contributes to the regulation of nearly half of all expressed genes in a tissue-specific manner and plays a major role in rhythmic physiological function, including blood pressure rhythms and renal excretion rhythms (refs. 9–17 and Figure 1). In the kidney, for example, animal studies have demonstrated that genes encoding proteins involved in xenobiotic metabolism as well as various sodium transport genes are regulated by the clock mechanism (18–20). In humans, a small study in healthy volunteers showed that protein levels of Na^+^-Cl^−^ cotransporter (NCC) and prostasin in urinary exosomes varied over a 24-hour period (21), consistent with what has been shown using mouse models.

The circadian system is thought to have evolved in order to provide an adaptive homeostatic advantage for organisms living on a planet with 24-hour periods of light and dark. The molecular clock components are highly conserved: humans share approximately 30% homology with the BMAL1 homolog in bread mold, for example (22). Given the 24-hour, 7-days-a-week nature of modern life, disruption of the circadian system is increasingly common (23). Circadian disruption is defined as misalignment between the endogenous circadian rhythms of our internal body clocks and the external environment. Circadian disruption can occur acutely: a shift of just 1 hour due to daylight saving time is associated with an increase in adverse cardiovascular events (24). Epidemiological studies in this century have demonstrated that chronic circadian disruption, such as what occurs in shift workers, is a key risk factor for a number of pathologies, including cancer and cardiovascular disease. For example, shift workers have an increased risk of developing chronic kidney disease (25) and hypertension (26). Long working hours can cause circadian disruption and are associated with declining renal function (27). A recent meta-analysis demonstrated a link between long-term night shift work and increased systolic blood pressure (28). Sleep disorders, such as obstructive sleep apnea, are associated with both hypertension and non-dipping hypertension (29). There also seems to be a genetic relationship between single-nucleotide polymorphisms (SNPs) in circadian clock genes and the prevalence of some diseases. A genome-wide association study of 1304 individuals across 424 British families revealed BMAL1 haplotypes that are associated with the hypertension phenotype (30). SNPs in clock genes are associated with phenotypic variance in systolic blood pressure (31).

In this Review, we describe evidence linking circadian disruption to the pathologies of chronic kidney disease, lupus nephritis, aging, and hypertension. We also discuss the limitations of our understanding of the molecular mechanisms underlying these pathological states. It is important to note that the relationship between circadian disruption and disease states is bidirectional. In some cases, we do not know what comes first, the circadian malfunction or the disease state. However, we do know that circadian disruption and disease states constitute a vicious cycle in which each condition exacerbates the other. Finally, we discuss possible circadian-based interventions that might provide a benefit for renal function.

## Circadian disruption in diseases of the human kidney

### Chronic kidney disease.

Chronic kidney disease (CKD) is defined as damage to the kidneys or decrease in renal function that is sustained over at least 3 months. CKD is associated with disordered circadian rhythms of sleep, blood pressure, and proteinuria. In human subjects, worsening renal function is associated with later onset, shorter duration, and increased fragmentation of sleep (32). Consistent with these effects on sleep being due to kidney disease per se, rather than the effect of comorbidities, animal models of kidney disease, including subtotal nephrectomy in rats (33) and adenine model in mice (34), demonstrate abnormalities in sleep and activity. On the other hand, in patients with type 1 diabetes, circadian disruption precedes microalbuminuria or other clinical evidence of kidney disease (35). Similarly, mice with genetic deletion of the circadian CLOCK protein are more vulnerable to adenine-induced CKD than wild-type mice (34). These observations suggest that circadian dysfunction might predispose to kidney disease. Thus, CKD and circadian dysfunction might have a bidirectional relationship, with circadian disruption accelerating kidney dysfunction and kidney disease causing abnormalities in circadian rhythms.

CKD is also associated with non-dipping pattern of blood pressure, suggesting disrupted circadian rhythms of blood pressure. Clinically, non-dipping is defined as less than 10% difference between night and day blood pressure. The prevalence of non-dipping hypertension in CKD varies from 60% to 80% and increases with worsening kidney function (36). In patients with CKD, non-dipping pattern of blood pressure is independently associated with an increased risk of death or progression to end-stage renal disease (37). In a small study of 20 patients with end-stage renal disease who received kidney transplantation and had ambulatory blood pressure monitoring performed before and a year after the transplant, the percentage of patients who were considered dippers rose from 15% at 1 month before transplantation to almost 40% a year after transplantation (38). The dipping status was influenced by the renal function after transplantation and the use of immunosuppressive medications. Although the data on whether nighttime dosing of antihypertensive medication reduces nocturnal blood pressure or restores nocturnal dipping are inconclusive (39–41), a randomized controlled trial of 661 patients with CKD, in which subjects were randomized to receive at least one of their antihypertensive medications at night, found that the risk of cardiovascular events was one-third in comparison with those who received all their medications in the morning (42). The reduction in cardiovascular events seemed out of proportion to that expected from the modest decrease in blood pressure, suggesting the possibility that restoration of circadian rhythms could have blood pressure–independent effects on the cardiovascular system.

The renin-angiotensin-aldosterone system is upregulated in CKD. Angiotensin II acting via its cognate receptor AT1 modulates the activity of the suprachiasmatic neurons in vitro (43). Whether AT1 activation modulates the central circadian clocks in vivo and contributes to circadian dysfunction in CKD is unknown. Further, gut dysbiosis and accumulation of uremic toxins in CKD might disrupt the circadian clock. The aryl hydrocarbon receptor, an environmental sensor that can bind uremic toxins, demonstrates rhythmic expression and forms a heterodimer with BMAL1 to modulate expression of circadian genes (44, 45). However, whether the aryl hydrocarbon receptor contributes to circadian dysfunction in CKD remains to be determined.

CKD is associated with the development of long-term complications such as hyperphosphatemia, secondary hyperparathyroidism, vascular calcification, accelerated atherosclerosis, bone mineral disorders, and anemia. Serum phosphate levels exhibit a circadian rhythm in healthy individuals (46). While the afternoon peak is attenuated by a low-phosphate diet, the nocturnal peak appears to be independent of diet and modulated by unidentified endogenous factors. This raises the intriguing possibility that the circadian system might regulate serum phosphate levels. Although reports of altered rhythmicity of serum phosphate with CKD in human subjects are variable, a recent study of mice with adenine-induced kidney disease suggests that circadian rhythmicity of phosphate is disrupted only by a high-phosphate diet and induction of vascular calcification (47). However, the molecular mechanisms underlying these observations remain to be elucidated, and whether circadian disruption can lead to vascular calcification needs further study. Similarly, circadian rhythmicity has been observed in the levels or activity of most hormones involved in bone mineral metabolism (48, 49). Erythropoietin levels (50), reticulocyte counts (51), and parathyroid hormone (52) all exhibit circadian rhythmicity in human subjects. However, the role of circadian dysregulation in contributing to the development of long-term complications of CKD remains to be studied.

**Figure 2:**
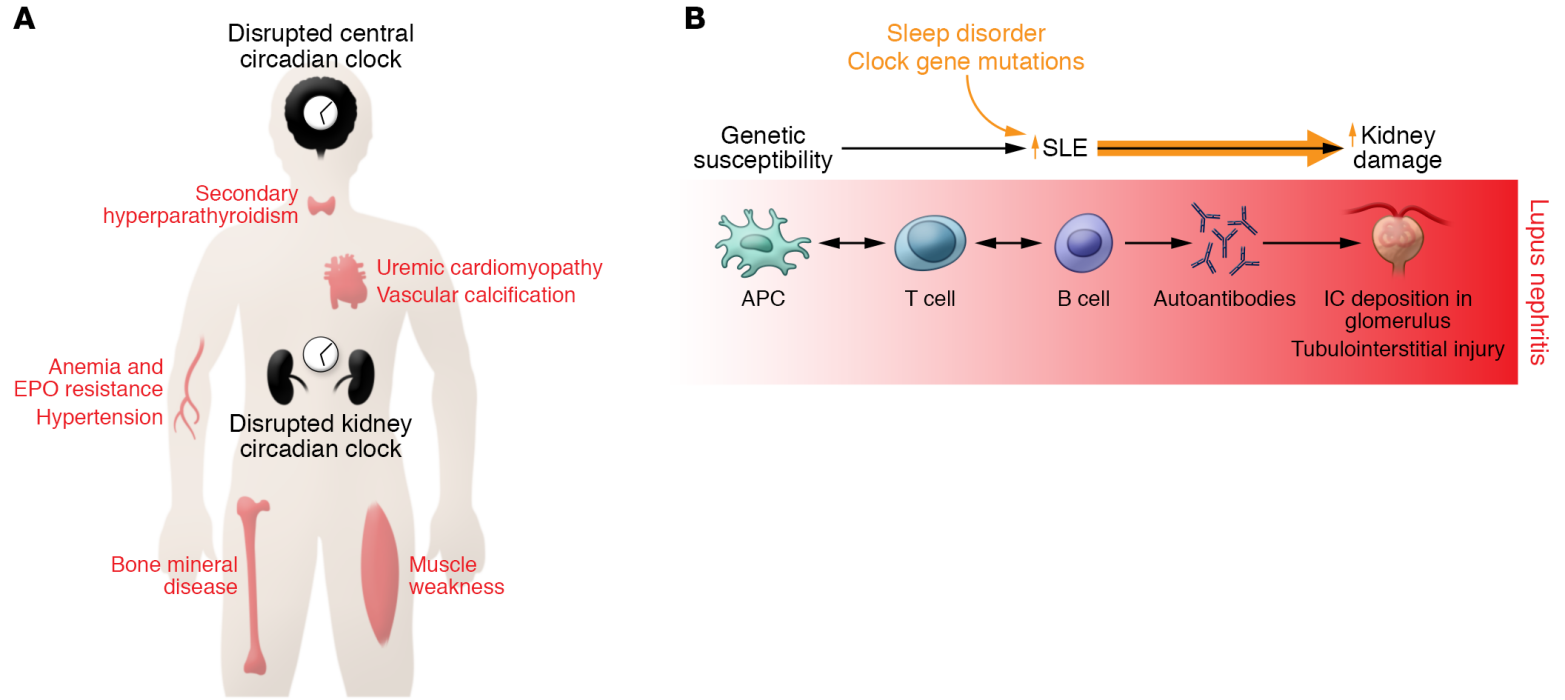
Disruption of circadian rhythms in disease state. (**A**) Complications of CKD. Kidney disease is associated with disruption of peripheral and central circadian rhythms. The molecular clock modulates the levels or activity of serum phosphate, parathyroid hormone, erythropoietin (EPO), and other hormones that are known to exhibit diurnal rhythms. (**B**) Schema of progression of SLE to end-organ renal disease (lupus nephritis [LN]) and potential contribution from disturbed circadian clock. Genetically susceptible individuals develop SLE. During disease progression there is a complex crosstalk between multiple cell types involving both innate and adaptive immune systems. The antigen-presenting cells (APCs) present self-antigens from various sources to T lymphocytes, which results in generation of autoreactive T cells. These CD4^+^ T lymphocytes in turn instruct B cells to produce autoantibodies of different specificities that deposit as immune complexes (ICs) in the glomeruli. This leads to progressive glomerular pathology and local production of chemoattractants and matrix proteins, resulting in immune cell infiltration and tissue damage. Loss of glomerular permeability also leads to tubulointerstitial injury, which is perpetuated by intrinsic tubular cell inflammatory phenotype and infiltrating immune cells and eventually leads to renal failure. Sleep fragmentation or genetic mutations in key clock proteins in SLE patients can potentially accentuate immune cell effector function. Furthermore, mutations in the renal intrinsic cells’ clock genes can render them susceptible to injury, as local injurious events unfold during the progression of LN.

The signaling mechanisms that are perturbed in CKD can conceivably disrupt the kidney clock. However, disrupted sleep in human subjects with CKD and experimental evidence for disturbed activity and central clock output from animal studies discussed earlier suggest that the central clock in the suprachiasmatic nucleus is perturbed in kidney disease as well. This circadian dysfunction is likely to exacerbate the known complications of CKD (Figure 2A). Although the evidence for circadian dysfunction in CKD is compelling, the molecular mechanisms involved in the pathogenesis remain largely unknown.

### Lupus nephritis.

Although sleep disruption and fatigue are prominent complaints in patients with systemic lupus erythematosus (SLE) and negatively affect quality of life (53–57), the role of the circadian clock in SLE and lupus nephritis (LN), a common complication in patients with SLE, has not been explored in detail. We briefly discuss the general pathobiology of LN and summarize the current knowledge linking disturbances in clock cycle and SLE.

SLE is an autoimmune disease of unknown etiology that mainly affects women of reproductive age. LN is the most common end-organ manifestation of SLE and is the major cause of morbidity and mortality (58, 59). Immune complex deposits, composed of antinuclear, anti-C1q, and cross-reactive antiglomerular autoantibodies (60–64), are found in the glomeruli (65, 66) and are considered the most common initiators of renal disease in SLE. This leads to local production of cytokines and chemokines that recruit leukocytes to perpetuate renal injury (67–69). The T and B lymphocytes from LN kidneys are clonally expanded, and the same T cell clones have been detected in the peripheral blood (70, 71). Macrophages infiltrate LN kidneys, and this is associated with poor outcomes (72–74). These intrarenal innate and adaptive immune responses can synergize with systemic autoimmunity and worsen overall outcomes. Although the circadian clock has been shown to regulate immune responses and macrophage function, the role of the circadian system in the development of SLE or LN has not been explored in detail.

Sleep disturbances are commonly reported in SLE patients (56, 75, 76) and negatively affect quality of life (57). Early studies revealed that in patients with SLE, levels of circulating immune complexes drop during sleep, supporting a role for the circadian system in the regulation of immunological processes in SLE (77). The association between sleep quantity and incidence of SLE (78) was studied in a cohort of individuals at increased genetic risk for SLE (79) who did not meet diagnostic criteria for SLE (80) at their baseline visit. In a follow-up study using questionnaires and chart review, less than 7 hours of sleep per night was independently associated with increased risk of developing SLE, suggesting a possible causal role for sleep disturbances and perhaps circadian dysfunction in pathogenesis of SLE (78). In patients with chronic glomerulonephritis and SLE who were treated with the glucocorticoid prednisolone, the nighttime dip in blood pressure was lost, with the blood pressure lowest in the afternoon, rising throughout the night, and peaking in the morning (81). Additional studies are needed to evaluate the role of this circadian disruption in disease progression.

Dysregulated immune responses are a cardinal feature of SLE/LN, and immune cells display a circadian effector pattern. Perturbations in immune cell circadian clock can influence outcomes of SLE/LN. In humans, natural regulatory T cell (Treg) number and function follow a rhythm across a 24-hour period. Sleep deprivation abrogated this rhythm and impaired Treg function (82). These findings are noteworthy, as Tregs are dysregulated and functionally impaired in SLE (83, 84), raising the possibility that alterations in the circadian clock in SLE could impair Treg functions.

Macrophages are the harbingers of renal disease in LN and have been implicated in the pathology of several spontaneous and induced models of murine LN (73, 85–87). It is well documented that a circadian clock in macrophages controls their inflammatory responses as well as mitochondrial metabolism (88–91). The circadian clock protein BMAL1 regulates IL-1β in macrophages via nuclear factor erythroid 2–related factor 2 (Nrf2) (90). Nrf2 expression is increased in glomeruli of patients with LN, and in mouse models of LN Nrf2 suppresses LN through inhibition of oxidative injury and the NF-κB–mediated inflammatory response (92). Similarly, BMAL1-knockout macrophages are unable to sustain mitochondrial function, and display enhanced glycolysis as well as HIF-1α–dependent metabolic reprogramming and inflammatory response (89). In an animal model of LN, renal macrophages switch toward glycolysis, secrete more IL-1β, and recruit neutrophils to damage the kidneys (93). Collectively, these findings pose several pertinent questions, like how local microenvironment dictates the function of macrophage molecular clock, particularly in LN.

Few studies have examined the effect of genetic deletion of clock proteins in mice with spontaneous or drug-induced lupus. Mice deficient in the circadian clock genes *Cry1* and *Cry2* have elevated serum IgG concentrations, serum antinuclear antibodies, and precipitation of IgG, IgM, and complement in glomeruli (94). This study also showed that CRY deficiencies enhance the rate of B cell maturation and stimulate B cell developmental in the spleen and peritoneal cavity, leading to an increase in autoantibody production. Another study, by Palma et al., showed that NZB/W mice (spontaneous model of LN) subjected to sleep deprivation exhibited an earlier onset of the disease, as reflected by the increased number of antinuclear antibodies, though the severity of the disease based on proteinuria and survival data was comparable (95). In contrast, using the same strain of mice, Mishra et al. recently reported significant differences in the renal expression of circadian clock–associated genes and proteins in young and nephritic mice (96). The kidneys of young NZB/W mice displayed a normal circadian pattern of expression of *Bmal1*, *Clock*, *Per*, and *Cry* genes compared with the nephritic mice. There was also a significant reduction in BMAL1 protein expression at various time points in nephritic NZB/W mice compared with young mice.

The finding that the disturbances in circadian rhythm and dysregulated expression of key clock genes and proteins are associated with progression of SLE and LN is becoming well documented. Collectively, genetic mutations and sleep disturbance may synergize with systemic and local inflammation to worsen LN (Figure 2B). Nevertheless, there are limited reports regarding the role of the circadian clock in progression of LN or other end-organ pathology associated with SLE. Given the known link between activation of the proinflammatory transcription factor NF-κB and lupus (97), it is interesting to note that NF-κB has been linked to circadian disruption and altered clock gene expression in response to inflammation (98). A recent report showed that circadian disruption during inflammation could be ameliorated by NF-κB inhibition in a mouse model of obesity (99), raising the question of whether NF-κB inhibition could affect circadian disruption in LN. Whether disruptions in circadian rhythm affect SLE manifestations and whether the circadian clock is a target for treatment of lupus and its associated morbidities remain to be determined.

### Hypertension.

Because the kidneys play a critical role in blood pressure regulation, hypertension can be both cause and consequence of renal damage. Circadian disruption is likely to exacerbate these effects since it is known to negatively affect human health. Shift work is becoming more common in the 21st century (28). It is estimated that about 20% of workers in Europe and the United States have a shift work schedule, defined as something other than a “9 to 5” schedule. Shift work is associated with various adverse health effects, including increased prevalence of hypertension. A meta-analysis of 45 studies, including a total of 117,252 workers, found that there was a significant increase in both systolic and diastolic blood pressure in permanent night workers (28). Rotational shift work was associated with a significant increase in systolic blood pressure only. There was no significant association between shift work and hypertension in this study, but the average worker age was below 40 years old. Data from Korea showed that among factory workers taking antihypertensive medications, blood pressure control was worse in night shift workers compared with day shift workers (OR 0.74, 95% CI 0.68–0.80; ref. 100). This finding remained significant even after adjustment for age, sex, obesity, exercise, smoker status, and alcohol intake. Studies like these demonstrate the risks associated with disruption of normal circadian patterns of behavior and physiology.

Many patients diagnosed with essential hypertension have a normal day/night pattern of blood pressure rhythms, but the overall mean arterial pressure is shifted up. Hypertensive patients with a reduced day/night rhythm of blood pressure are classified as having non-dipping hypertension. The prevalence of non-dipping hypertension is difficult to estimate since it requires the use of 24-hour ambulatory blood pressure monitoring. It is of paramount importance to increase the use of this method, however, as non-dipping hypertension is a critical risk factor for adverse cardiovascular outcomes (reviewed in ref. 101). Early studies demonstrated that restricting dietary sodium could restore the nocturnal dip of individuals with sodium-sensitive blood pressure (102). Patients with essential hypertension and an intact blood pressure dip at night maintain urinary sodium excretion rhythms on a high-salt diet, whereas patients with a non-dipping phenotype lose their rhythms of urinary sodium excretion (103). Sodium restriction significantly improved the rhythms of both urinary sodium excretion and blood pressure in the non-dipping hypertension group, but had no effect on the dipper group.

Timed urine collections provide an opportunity to assess circadian disruption in a noninvasive manner. The loss of the nighttime dip in blood pressure has been linked to disruption of rhythms in urinary sodium excretion (103). It should be noted that a recent study in mice showed that the blood pressure rhythm could be inverted by restriction of food intake to the inactive period, and this effect occurred in the absence of changes in the diurnal pattern of urinary sodium excretion (104). In a pathophysiological setting, it was recently shown that restricting food intake to the active period restored the normal blood pressure rhythm in non-dipping, diabetic mice (105). In a clinical study, 642 Chinese adults with primary hypertension underwent 24-hour ambulatory blood pressure monitoring, and 24-hour urine sodium excretion was measured along with morning urine sodium concentration (106). Both 24-hour urinary sodium excretion and morning urinary sodium concentration had a positive association with blood pressure. Healthy individuals excrete more sodium during the day than during the night. In a cross-sectional study with over 1000 patients in Switzerland, Del Giorno et al. paired 24-hour ambulatory blood pressure data with night and day urine collections to assess the relationship between blood pressure dipping, day/night ratios in sodium excretion, and age (107). Patients with the lowest ratio of day/night sodium excretion tended to be older (>50 years of age) and have higher nighttime blood pressure (5–6 mmHg). These findings support the idea that the loss of renal excretion rhythms is associated with aging and may negatively affect blood pressure dipping. This study clearly demonstrates the utility of using timed urine collections as a way to assess rhythmic renal function. Given the strong association between renal sodium excretion and blood pressure, this method could also provide a means to identify those at risk for hypertension-associated end-organ damage.

### Aging.

It is well established that kidney function decreases with increasing age (108–110). Age-related renal structural changes are observed independent of comorbidities. Kidney mass declines after age 50, with an accompanying decline in the number of functional glomeruli and nephrons (111–113). The incidence of both nephrosclerosis and glomerulosclerosis is significantly higher in patients above 70 years old, even when examining biopsies from normotensive individuals (114–117). The structural changes that occur in the aging kidney are accompanied by alterations of renal physiology. Glomerular filtration rate (GFR) declines with increasing age, but the rate at which GFR decreases with age is still uncertain, as different studies report varying degrees of GFR decline (111, 118–122). A decrease in renal blood flow is also observed with increasing age, which is also associated with an increase in renal vascular resistance and filtration fraction (123, 124). Dysregulation of electrolyte handling has also been observed among the elderly. Older adults have reduced lithium clearance compared with young subjects, despite the fact that the sodium excretion is similar (123). This suggests that proximal tubule reabsorption of sodium seems to increase with age, but distal nephron sodium reabsorption decreases, which could contribute to the higher rate of salt-sensitive blood pressure among the elderly (125, 126).

In addition to overall changes in kidney structure and function, circadian rhythms in behavior, sleep/wake cycle, and other physiological parameters are negatively affected in older populations (127). Changes in sleeping patterns are extremely common among aging adults (128). As age increases, the sleep/wake cycle shifts and individuals tend to fall asleep and wake up earlier than younger individuals (129, 130). Cortisol, which has been demonstrated to regulate rhythms in peripheral clocks, has an altered rhythm of production in older adults (131, 132). Nighttime cortisol seems to increase with age in both men and women, but an increase in the morning acrophase of cortisol is observed only in women (133). These changes in cortisol are likely related to the age-related sleep cycle changes, but may also affect renal rhythms. Multiple studies in varying aging populations have demonstrated that renal diurnal rhythms deteriorate with age. Electrolyte excretion rhythms have been shown to dampen with increasing age (134). Compared with patients in the 25 to 35 age range, healthy subjects in the 60- to 80-year-old group had a reduced day-to-night ratio of water, electrolyte, and solute excretion (135). Additionally, older individuals showed reduced 24-hour sodium and potassium excretion, despite solute excretion and urine volume similar to those in the younger group.

A common symptom associated with aging is nocturia, which is defined as waking up one or more times at night to urinate (136). The prevalence of nocturia increases with age in both men and women, with around 60% of people older than 70 waking up two or more times nightly to urinate (137, 138). Nocturia pathophysiology in the elderly involves many different factors, but significant contributions arise from age-related changes in the kidney, such as reduced ability to concentrate urine (139) and the inability to excrete solutes shortly after a meal (140). Compared with age-matched non-nocturic patients, older nocturic patients not only had larger nighttime urine volume, but also excreted significantly more sodium, chloride, and potassium at night (141, 142). The nocturic patients excreted half of their total 24-hour sodium within the nighttime period, whereas the non-nocturic patients excreted twice the amount of sodium during the daytime compared with the nighttime. No differences were observed in urine aldosterone excretion rates between the two groups. Furthermore, decreased day-to-night ratios (altered diurnal rhythms) of diuresis in older men with nocturia are associated with higher nighttime mean arterial pressure (143). The alteration of circadian rhythms in patients with nocturia is associated with sleep disturbances, depression, arterial hypertension, and increased mortality and morbidity (144, 145). Despite the negative effects of nocturia, often patients do not report their symptoms to their physician because they consider nocturia to be part of the normal aging process (146).

Clinically, it is critical to determine the mechanisms behind dysregulation of the circadian clock in the aging kidney to improve quality of life and treatment strategies. For example, in a retrospective study using the US Renal Data System Dialysis Morbidity and Mortality Waves III/IV database, researchers examined the impact of the time of day at which dialysis was administered in a population of patients older than 60 years (147). Elderly patients who underwent dialysis in the morning or in the evening had a lower risk for mortality compared with those who received dialysis in the afternoon. Interestingly, time of day of dialysis did not seem to have an effect on mortality in patients younger than 60. These results are similar to those of an earlier study that reported increased survival of elderly end-stage renal disease patients who underwent dialysis in the morning versus the afternoon (148). Interventions and lifestyle changes such as exercise (149) have been hypothesized to help maintain renal rhythms, but this is a poorly researched area that could greatly benefit from additional studies. Additionally, the mechanisms that contribute to altered circadian rhythms in the elderly are still poorly understood and warrant further research. DNA methylation and histone modifications change with age, primarily in response to environmental stimuli as shown by studies with monozygous twins (150). These epigenetic changes may contribute to altered circadian rhythms in the elderly.

## Circadian therapeutics and the future

It is well established that loss of circadian rhythms in physiological functions can lead to disease. The evidence that this phenomenon occurs in patients with kidney disease is growing. As basic research brings us closer to understanding the pathophysiological mechanisms by which circadian disruption causes disease, the potential for circadian rhythm–based interventions is increasing. Using timed light therapy as an intervention, one small randomized controlled trial found that night shift workers had improved nighttime blood pressure dip with treatment. This increase in the nighttime blood pressure dipping status was associated with a decrease in serum glucose during oral glucose tolerance testing. There were no changes in serum insulin, melatonin, or cortisol, but plasma catecholamine levels were reduced (151). Early time-restricted feeding (all meals before 3 pm) in prediabetic men lowered blood pressure and increased insulin sensitivity (152). In a study including both male and female patients, a 10-hour self-selected window for eating resulted in lower blood pressure as well as a reduction in hemoglobin A_1c_ (153). In terms of treating patients with end-stage kidney disease, home dialysis and nocturnal dialysis offer attractive options for improving quality of life and potentially survival (154, 155). Whether some of the benefits are due in part to restoration of circadian rhythms is an intriguing possibility that merits further study. Preclinical rodent studies suggest that circadian clock modulatory compounds may have benefit in cardiovascular disease (156). There is growing interest in “drugging the clock,” which could include optimizing the timing of medication delivery as well as small molecules that influence clock protein function, in humans in several different diseases (157). With the increasing availability of circadian rhythm–based therapeutics, it is becoming more and more important that we gain a more complete understanding of the mechanisms by which the circadian clock system regulates physiological function and how circadian disruption contributes to pathophysiology.

**Figure 3:**
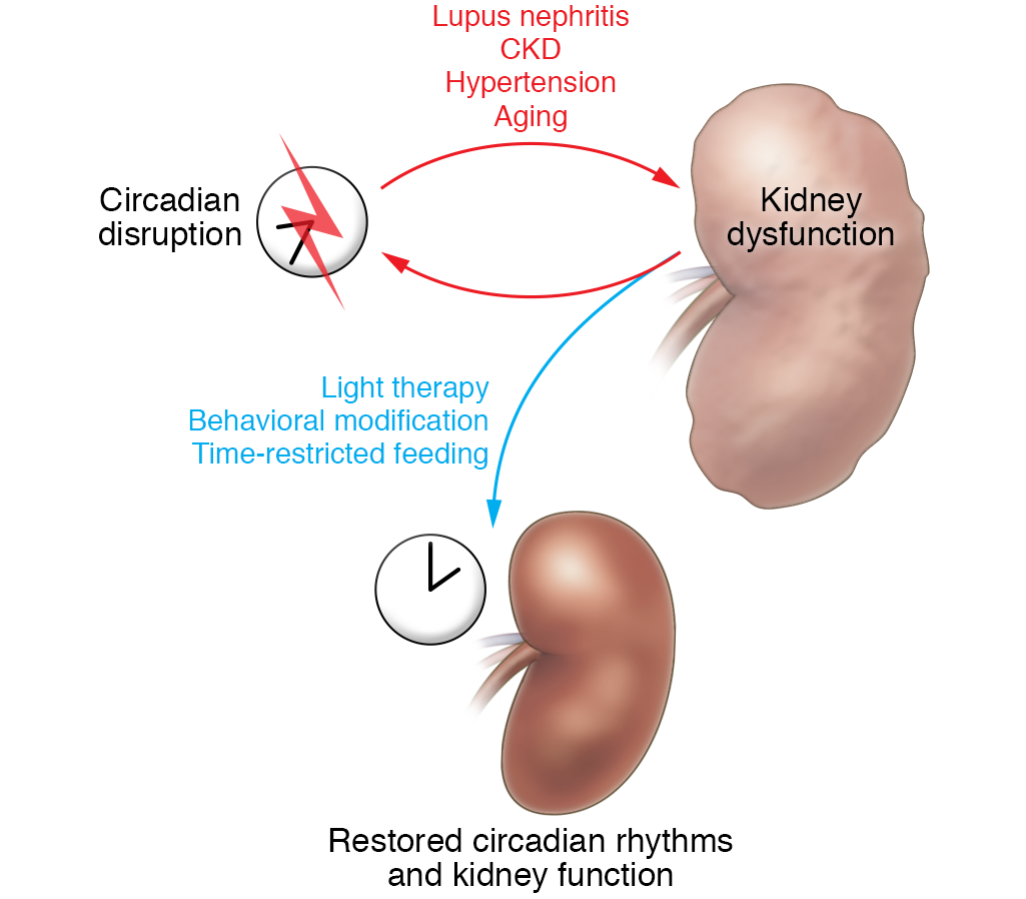
Proposed reciprocal relationship between circadian disruption and kidney disease. Circadian disruption may lead to renal disease, but kidney disease itself may cause circadian disruption. These pathological states may exacerbate each other. This vicious cycle may represent an opportunity for circadian rhythm–based interventions as novel therapies to restore circadian rhythms and physiological function.

In the future, adoption of circadian-based therapies will require development of tests to assay the extent of circadian disruption to facilitate diagnosis and assess response to therapy. The use of timed urine collections could shed light on the incidence of circadian disruption and help assess the effectiveness of circadian-based therapies in a noninvasive manner. Measurement of clock gene expression in blood samples may also be useful as a prognostic and diagnostic biomarker in patients on dialysis, those with CKD, or those with or at risk for acute kidney injury (158–160). Although the potential to use circadian principles in the treatment or management of kidney disease is growing (Figure 3), the mechanisms by which disrupted circadian rhythm mediates and perpetuates renal injury remain to be elucidated, as basic research in this area is still in the early stages. Given the physiological and clinical significance of circadian rhythms to human health, more detailed examination of the circadian clock in models of renal disease is necessary and is expected to bring a deeper understanding of renal versus systemic circadian homeostasis.

## Version 1 02/01/2022

Electronic publication
